# Identification and quantification of prosthetic mitral regurgitation by flow convergence method using transthoracic approach

**DOI:** 10.1186/1476-7120-7-7

**Published:** 2009-02-12

**Authors:** Stephane Arques, Caroline Leonnet, Emmanuel Roux, Jean-François Avierinos

**Affiliations:** 1 Department of Cardiology, Aubagne Hospital, Aubagne, France; 2 Department of Cardiology, La Timone University of Medicine, Marseille, France

## Abstract

The present case report illustrates the clinical applicability of the proximal isovelocity surface area (PISA) method in identifying, locating and assessing paravalvular prosthetic mitral regurgitation by transthoracic echocardiography.

## Background

There is now large evidence that the proximal isovelocity surface area (PISA) is a simple, reliable method for the noninvasive assessment of native mitral regurgitation by Doppler echocardiography. Despite some limitations [1], the PISA method is widely used in daily practice as well as in clinical trials for diagnostic, therapeutic and prognostic purposes [2,3]. However, its applicability is underecognized in the setting of paravalvular prosthetic mitral regurgitation [2,3]. The present case report illustrates the clinical usefulness of the PISA method in identifying and evaluating paravalvular prosthetic mitral regurgitation by transthoracic Doppler echocardiography.

## Case presentation

**Figure 1:**
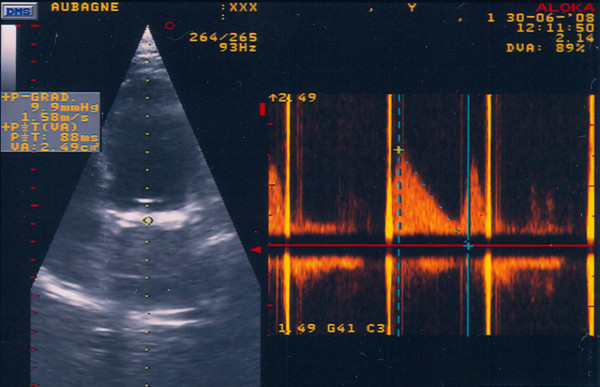
**Recording of the transprothetic mitral inflow by continuous wave Doppler in a 4-chamber apical view**. The peak early diastolic velocity was 158 cm/s, the mean gradient 3.5 mmHg and PHT 88 ms.

**Figure 2:**
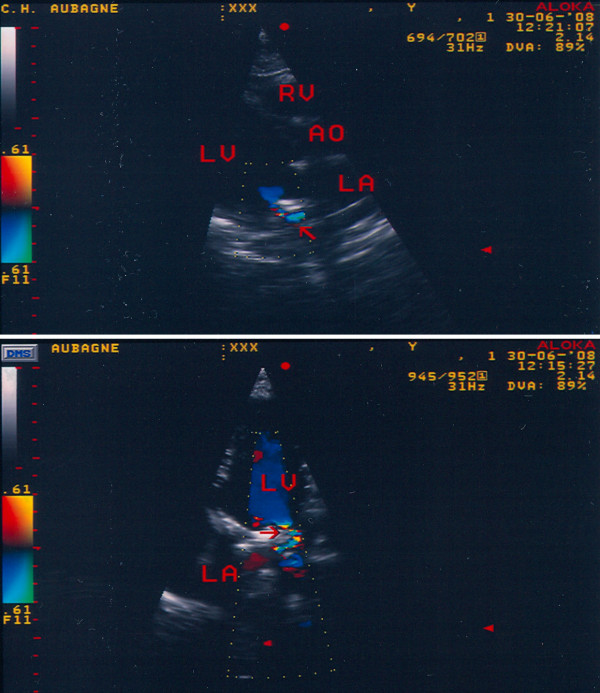
**Visualization of the paravalvular prosthetic mitral regurgitation (arrow) in parasternal long axis view (upper part) and 4-chamber apical view (lower part)**. AO: aorta; LA: left atrium; LV: left ventricle; RV: right ventricle.

**Figure 3:**
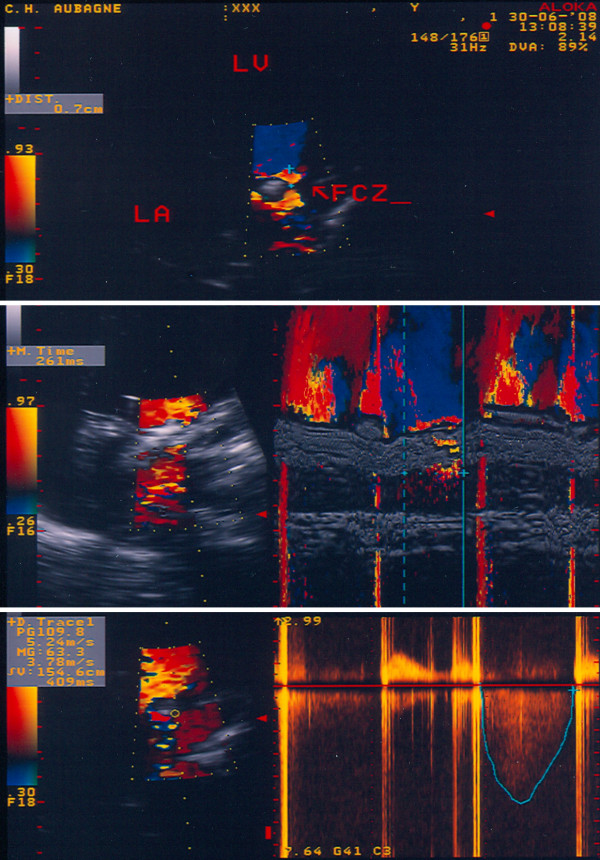
**Upper part: measurement of the radius (r = 0.7 cm) of the flow convergence zone (FCZ, arrow) at mid-systole in a 4-chamber apical view, with an aliasing velocity of 30 cm/s**. LA: left atrium; LV: left ventricle. Middle part: measurement of the regurgitation time (t, in secondes) by color M-mode at the level of flow convergence hemisphere; t = 0.261 secondes. Lower part: measurement of the velocity time integral (VTI, cm) and the peak regurgitant flow (Vmax, m/s) by continuous wave Doppler. VTI = 155 cm and Vmax = 5.2 m/s

Mr B., 61 year old, has been admitted at our institution for severe anemia. His medical history included mitral valve replacement (bileaflet mechanical valve) in 1993, diabetes mellitus, systemic arterial hypertension and vascular dementia. The patient was free of symptoms and signs of heart failure. Apical holosystolic murmur 2/6 was found at physical examination. The chest radiography did not show any abnormality. Sinus rhythm and left ventricular hypertrophy were present on the electrocardiogram. Blood sample analysis revealed severe anemia (5.5 g/dl). Transthoracic Doppler echocardiography was performed by an experienced cardiologist after informed consent was obtained. Left ventricular ejection fraction was 71%, left ventricular mass 115 g/m^2^, and there was no structural abnormality of the native aortic valve. Indexed left atrial volume was 29 ml/m^2 ^and the mean gradient accross the prosthetic mitral valve was 3.5 mm Hg. Peak early diastolic velocity of mitral filling was 158 cm/s and PHT 88 ms (Figure 1). A postero-lateral paravalvular prosthetic mitral regurgitation was identified by careful analysis of the prosthetic mitral valve both in parasternal and apical views (Figure 2). The peak instantaneous regurgitant flow rate (Qmax, ml/s), calculated as follows: Qmax = 2 × π × r^2 ^x Va where r was the radius of the flow convergence hemisphere at mid-systole (in cm) and Va the aliasing velocity (in cm/s), was 92 ml/s (Figure 3). The regurgitant volume (RV, ml), calculated as follows: RV1 = Qmax × t where t (in secondes) was the regurgitation time measured by color M-mode, was 24 ml (Figure 3). The regurgitant flow was recorded by continuous wave Doppler and the velocity time integral (VTI, cm) and the peak regurgitant flow (Vmax, m/s) were measured (Figure 3). The effective regurgitant orifice area (EROA, mm^2^) calculated as follows: EROA1 = (RV1 × 100)/VTI, was 15 mm^2 ^(Figure 3). The EROA calculated as follows: EROA2 = Qmax/Vmax, was 18 mm^2 ^(Figure 3). The RV, calculated as follows: RV2 = (Qmax × VTI)/(Vmax × 100), was 27 ml (Figure 3). All these parameters derived from PISA method were consistent with a mild to moderate prosthetic mitral regurgitation. The patient refused to give informed consent for transesophageal echocardiography. The diagnosis at discharge was hemolytic anemia related to mild paravalvular leak according to all the data collected during hospitalization.

## Discussion

The severity of paravalvular prosthetic mitral regurgitation is commonly assessed with simple Doppler indexes that indirectly reflect volume overload [4]. Bargiggia et al have first reported the usefulness of PISA method in identifying and locating prosthetic mitral regurgitation by transthoracic echocardiography [5]. The visualization of the flow convergence zone by thransthoracic echocardiography has been proposed by Cohen et al as a marker of significant prosthetic mitral regurgitation, however such a qualitative assessment suffers from lack of specificity [6]. With the steady improvement of the quality of Doppler echocardiographs, the PISA method presently offers the ability to provide useful information not only on identification and location but also on quantitative assessment of paravalvular prosthetic mitral regurgitation by transthoracic approach [7]. To our knowledge, only one clinical study has addressed the applicability of PISA method by transthoracic Doppler echocardiography in the setting of paravalvular prosthetic mitral regurgitation, which included 30 consecutive patients with mitral prosthesis (21 with mechanical valve and 9 with bioprosthesis). Four, 13 and 13 patients had mild, moderate and severe paravalvular prosthetic mitral regurgitation, respectively. Assuming the radius r and Qmax constant over the systole, RV was measured as follows: RV = (Qmax × t) × α/180, where α/180 was the angular correction applied in presence of parietal constraint. It is of clinical importance to emphasize that this alternative method offers the ability to overcome the difficulty of adequatly recording the regurgitant flow by transthoracic continuous wave Doppler in the setting of excentric jets. By this way, the feasibility of RV was high (93%), with an intra- and inter-observer variability of 6 and 8%, respectively. RV was a strong predictor of severe prosthetic mitral regurgitation with an optimal cut-off value of 53 ml (area under the ROC curve of 0.97 [0.82–0.99], p < 0.0001; sensitivity of 92% and specificity of 93%). The standard cut-off value of 60 ml used for defining severe native mitral regurgitation was 69% sensitive and 100% specific. Notably, the flow convergence zone could not be distinguished from the ejection flow in the left ventricular outflow tract in the 2 patients with moderate paravalvular leak located at the septal side of mitral prosthesis. The regurgitant flow by transthoracic continuous wave Doppler, and subsequently the EROA, were obtained in 63% of patients. EROA was a good predictor of severe prosthetic mitral regurgitation with an optimal cut-off value of 32 mm^2 ^(area under the ROC curve of 0.90 [0.68–0.98], p < 0.001; sensitivity of 91% and specificity of 75%). The standard cut-off value of 40 mm^2 ^used for defining severe native mitral regurgitation was 64% sensitive and 100% specific. The reliability of the PISA method has been later confirmed in this clinical setting by Vitarelli et al using transesophageal echocardiography [8]. 47 patients with mechanical valve were included, of whom 25 had severe paravalvular prosthetic mitral regurgitation. Qmax > 200 ml/s and EROA > 45 mm^2 ^were 96% sensitive and 90% specific for the prediction of severe mitral regurgitation. Nevertheless, the use of transthoracic echocardiography offers the ability to overcome the limitations inherent to other invasive methods for follow-up. Further studies will address the usefulness of new echocardiographic modalities, such as real-time three-dimensional color Doppler echocardiography, in this clinical setting [9].

## Consent

Written informed consent was obtained from the patient for publication of this case report and any accompanying images. A copy of the written consent is available for review by the Editor-in-Chief of this journal.

## Competing interests

The authors declare that they have no competing interests.

## Authors' contributions

SA performed Doppler echocardiography, participated in the design of the study and wrote the manuscript. CL and ER were involved in the patient's clinical care. JFA critically reviewed the manuscript and participated in the design of the study. All the authors read and approved the final manuscript.
